# Knowledge and attitudes about assisted reproductive technology: Findings from a Hungarian online survey

**DOI:** 10.1016/j.rbms.2021.06.005

**Published:** 2021-07-03

**Authors:** Ivett Szalma, Tamás Bitó

**Affiliations:** a Centre for Social Sciences, Hungarian Academy of Sciences Centre of Excellence, Budapest, Hungary; b Corvinus University of Budapest, Budapest, Hungary; c Department of Obstetrics and Gynaecology, University of Szeged, Szeged, Hungary

**Keywords:** Assisted reproductive technology, Knowledge gap, Attitudes towards ART, Postponement of childbirth

## Abstract

This study aimed to evaluate the general knowledge and attitudes about assisted reproductive technology (ART) and the influence of sociodemographic features on knowledge and attitudes in a large sample of men and women of reproductive age in Hungary. A cross-sectional online survey study was conducted among 1370 men and women between 18 and 50 years of age in Hungary. The questionnaire included questions about self-rated knowledge, an attitude item, and eight questions concerning general knowledge about ART. In addition, participants were asked sociodemographic background questions. The results show that approximately half of the respondents (49.3%) rated themselves as fairly knowledgeable about ART. However, 56% of the respondents answered just three of the eight knowledge questions correctly. Both men and women had limited knowledge about the success rate of ART, the costs of ART and the age limit to access ART. The greatest lack of knowledge about ART was about its risks: the majority of respondents did not know that in-vitro fertilization poses health risks for women and conceived children. Regarding attitudes, the majority of respondents had a very positive attitude towards ART. Only those respondents who were religious were less supportive of ART. These data suggest that men and women of reproductive age overestimate their ART-related knowledge. As most men and women would like to have biological children in Hungary, there is a critical need for public education.

## Highlights

• To evaluate the general knowledge and attitudes about assisted reproductive technology in Hungary.
• Despite the overly positive attitudes towards assisted reproduction, there is a lack of coherent knowledge about ART in Hungary.
• In particular, there is a knowledge gap about the risks of ART for conceived children and for women.

## Introduction

The number of children born through assisted reproductive technology (ART) has increased markedly since 1978, when the first baby conceived through in-vitro fertilization (IVF) was born. The reason for the spread of ART is partly related to the postponement of parenthood in most developed countries (Cheung et al., 2019, Mills et al., 2011). Women’s fecundity declines sharply with age after 35 years (Eijkemans et al., 2014, Hammarberg et al., 2013, Utting and Bewley, 2011). As in the USA, Canada, Australia and other European countries, the mean maternal age at first delivery is increasing in Hungary (Berrington and Pattaro, 2014, Bretherick et al., 2010, Cheung et al., 2019, Daniluk et al., 2012, Maheshwari et al., 2008). The mean age of mothers at first childbirth in Hungary was 23.4 years in 1995, and this had increased to 28.6 years by 2017 (HCSO, 2019). The postponement of parenthood is due to many factors, such as lack of a suitable partner (Szalma and Takács, 2015), conflicting work obligations (Mills et al., 2008, Dommermuth et al., 2017) and extended education (Beaujouan et al., 2016, Berrington and Pattaro, 2014). However, it is unknown whether women and men who delay their parenthood make this choice with the full knowledge of its consequences (Wyndham et al., 2012).

Studies have shown that the decision to have children is multifaceted, and is determined not only by individual, social and economic factors but also by social policies (Balbo et al., 2013; Mills et al., 2011). ART can also represent a potential policy lever for raising fertility rates in some pronatalist countries (Birenbaum-Carmeli, 2016, Präg and Mills, 2017, Szalma and Djundeva, 2019). For example, in Hungary, the Government announced the National Human Reproduction Programme, which is a comprehensive programme to support infertile couples. As part of the measures, six fertility clinics have been nationalized and their services (fertility diagnostics, medications and treatments) were made free of charge as of 1 July 2020 (Cseresnyés, 2019). This measure fits into the existing pronatalist family policy very well because the Government of Hungary expects it to result in the birth of 4000 babies per year (About Hungary, 2019). The investigation of attitudes and knowledge of the general public is crucial because the number of ART users in Hungary may be expected to increase due to the generous financial state support of ART.

Previous studies (Adashi et al., 2000, Bretherick et al., 2010, Bunting et al., 2013, Daniluk et al., 2012, Daniluk and Koert, 2013, Hammarberg et al., 2013, Harper et al., 2017, Maheshwari et al., 2008; Pedro et al., 2018; Peterson et al., 2012, Utting and Bewley, 2011, Wyndham et al., 2012) have reported that there are misconceptions among women about their own fertility, the risks of a pregnancy at an advanced age and the effectiveness of ART. Many women in Hungary wrongly believe that ART will work until menopause (Szalma, 2021, Vicsek, 2018). Although technological options can help to a degree with age-related fertility issues, they cannot compensate completely for the drop in fertility rate (Leridon, 2004, Liu and Case, 2011) as the success rate of ART declines among women aged > 40 years (Utting and Bewley 2011). O’Brien et al. (2017) found that the rate of live births after ART was 33.3% among women aged 30–35 years, and 14.8% among women aged 40–44 years. Moreover, not only is the success rate (pregnancy rate) fairly low, but the effectiveness is usually limited, which means that couples have to go through an average of three complete cycles in order to achieve a pregnancy (Stewart et al., 2011, Wyndham et al., 2012).

However, ‘social egg-freezing’ can it make possible for women to have their own biological children at an advanced age (Meissner et al., 2016, Sándor et al., 2018). ‘Social’ refers to the idea that women decide to postpone childbirth voluntarily because of difficulties such as finding the right partner, work obligations or extended education. Women and men who have children at a later age than the mean age are considered ‘postponers’. In Hungary, higher-educated women are more likely to be postponers (HCSO, 2019). In order to increase their chances of establishing pregnancies and having healthy babies, it is better if these women cryopreserve their eggs at a younger age, at least before 35 years (Mertes and Pennings, 2011). Social egg-freezing is not available in Hungary, unlike other European countries such as Denmark, the UK (Lallemant et al., 2016) and Germany (Meissner et al., 2016).

As well as the availability of social egg-freezing, the age limit for participation in ART also differs between European countries (Präg and Mills, 2017). Most European countries have a limitation on ART based on women’s age, and some countries also apply a limitation based on men’s age. In Hungary, the age limit for women to have access to ART is 45 years, but there is no age limit for men (Präg and Mills, 2017). In focus group discussions with temporarily childless women aged 18–35 years, the participants said that they can accept the age limit for women but were surprised to find out there was no similar limit for men (Szalma, 2021). They considered it unfair that men’s age is not regulated, and they believed that this was mainly due to the idea that taking care of children is primarily the responsibility of women (Szalma, 2021).

Public attitudes in the USA as well as in many other Western countries are overly positive towards ART (Meissner et al., 2016, Szalma and Djundeva, 2019, Wennberg et al., 2016, Wyndham et al., 2012), which has emerged in public discussions as a measure that can help couples facing infertility (Payne and Korolczuk, 2016, Shlomo and Kabizon-Pery, 2019, Utting and Bewley, 2011). Consequently, its risks are rarely in the focus of public discussions of those who have not participated in ART. However, ART presents various risks to both women and their conceived children (Hansen et al., 2005, Rebar, 2013, Wyndham et al., 2012).

Not only do attitudes towards ART and its legislation differ between countries in Europe, but its costs vary widely. Some countries offer complete coverage through national health plans, and other countries offer no compensation at all. Most countries in Europe offer partial coverage (Keane et al., 2017). Hungary offers complete coverage through national health plans, but certain costs of ART (e.g. a considerable part of the cost of gonadotrophic drugs) were being paid by the users when this research was conducted (Keane et al., 2017). However, this changed on 1 July 2020, when the state began to cover ART-related medicine fully in Hungary (Cseresnyés, 2019).

Regarding the effect of sociodemographic variables, previous studies have reported a gender gap as women had more knowledge about general fertility and ART than men (Bunting et al., 2013, Hammarberg et al., 2013, Meissner et al., 2016, Stoebel-Richter et al., 2012). Several studies were conducted among university students (Bunting et al., 2013, Hashiloni-Dolev et al., 2011, Hickman et al., 2018, Meissner et al., 2016, Peterson et al., 2012), making it difficult to evaluate knowledge related to ART among older populations and among people of reproductive age with a lower level of education. However, in certain population-based studies, researchers did not find significant associations between age and fertility awareness (Daniluk et al., 2012, Daniluk and Koert, 2013, Daumler et al., 2016, Garcia et al., 2016, Maeda et al., 2015), while other studies found that older people had higher fertility awareness than younger participants (Bunting et al., 2013, Garcia et al., 2016). In a systematic review on fertility awareness by Pedro et al. (2018), similar mixed results were found about the role of education in fertility knowledge. Some researchers found no association (Daniluk and Koert, 2013, Daumler et al., 2016), while others found significantly positive associations between education level and fertility awareness (Bunting et al., 2013, Daniluk and Koert, 2013, Garcia et al., 2016, Hammarberg et al., 2013, Meissner et al., 2016, Stoebel-Richter et al., 2012).

However, while several studies focusing on general fertility awareness have been published, papers that investigate awareness about ART are less widespread. As such, the aim of this study was to examine knowledge about ART and attitudes towards ART among highly educated males and females in Hungary. The questions that guided this research formed the following seven hypotheses: H1, approximately half of the respondents would overestimate the success rate and effectiveness of ART; H2, the majority of respondents would know that cryopreservation (egg-freezing) before 35 years of age can significantly prolong a woman’s fertility; H3, most respondents would be aware of the ART age limit for women in Hungary, but most would not be aware that there is no ART age limit for men in Hungary; H4, most respondents would have very little knowledge about the risks of ART; H5, most respondents would have some knowledge about the costs of ART; H6, women, respondents who are better educated, respondents who belong to older age groups, parents and respondents who have previous experience with ART would have more knowledge about ART than men, respondents who have a lower level of education, respondents who belong to older age groups, childless respondents, and respondents who do not have any experience with ART; and H7, respondents who classified themselves as religious would be the least likely to have positive attitudes towards ART.

## Materials and methods

### Questionnaire design

To measure knowledge about ART and attitudes towards ART, the Hungarian version of the Fertility Awareness Survey (FAS) was applied. The questionnaire, originally developed by Daniluk et al. (Daniluk et al., 2012, Daniluk and Koert, 2013), consists of 16 knowledge questions for women (FAS), and partially overlaps with 20 knowledge questions for men from the Fertility Awareness Survey-Male (FAS-M). As we wanted to involve both genders in our research, the two questionnaires were unified; additionally, some of the items were also adjusted to the Hungarian context.

This questionnaire consisted of three parts: the first part asked about self-rated levels of knowledge about fertility and ART, and respondents’ attitudes towards ART. Respondents were asked to rate their current level of knowledge about ART on a four-point scale of whether they have no knowledge (1), have some knowledge (2), are fairly knowledgeable (3) or are very knowledgeable (4). Participants had to indicate their attitudes towards ART on a five-point scale: oppose very strongly (1), oppose (2), neither oppose nor support (3), support (4) or support very strongly (5). The second part involved 17 and eight questions on knowledge about general fertility and ART, respectively. Each of the items contained a statement, and the respondents were asked to decide whether the statement was true or false, and rate their decision on a five-point scale (i.e. 1 = definitely not true, 2 = probably not true, 3 = do not know, 4 = probably true or 5 = definitely true) (Table 1). This paper only reports on the ART items.

**Table 1:** Self-rated knowledge and attitudes by gender.

Self-rated knowledge about general fertility	Male %	Female %	Self-rated knowledge about ART	Male %	Female %	Attitudes towards ART	Male %	Female %
Very knowledgeable	26	37.3	Very knowledgeable	11.9	16.8	Support very much	65.1	67.4
Fairly knowledgeable	61.8	56.1	Fairly knowledgeable	33.7	34.7	Support	25	23.6
Some knowledge	11.2	5.8	Some knowledge	45.2	41.5	Neither oppose nor support	7	6.4
No knowledge	1.1	0.8	No knowledge	9.3	7	Oppose	2.4	2.4
						Oppose very much	0.5	0.3
Total	100	100		100	100	100	100	100

ART, assisted reproductive technology.

*n* = 1370.

Half of the items (4, 5, 6 and 7) were imported from the FAS (Daniluk et al., 2012), while Item 8 was imported from the FAS-M (Daniluk and Koert, 2013). The first item was modified slightly in order to map whether the participants were familiar with the very low success rates of ART among women aged > 40 years. For this item, the following answer categories were applied: 1 = the success rate is much lower than 50%, 2 = the success rate is somewhat lower than 50%, 3 = do not know, 4 = the success rate is somewhat higher than 50%, and 5 = the success rate is much higher than 50%. The second item concerned the total cost of a single cycle of IVF, and half of the price was applied as a milestone to estimate whether the cost was under or over this figure. The answer categories provided were as follows: 1 = it costs much less, 2 = it costs somewhat less, 3 = do not know, 4 = it costs somewhat more and 5 = it costs much more. Items 3 and 8 measured knowledge about the upper age limit of access to fertility treatments for women and men, respectively, in Hungary. The third part of the questionnaire contained six questions regarding the sociodemographic features of the respondents (gender, age, education, religiosity, number of children, previous ART experience).

### Data collection

Participants were recruited via an online survey on a voluntary basis. We wrote an article about family policies in Europe on one of the most popular news portals in Hungary (quibit.hu) in February 2019, and asked the readers to fill out the questionnaire below the article if they were between 18 and 50 years of age. In the short introduction, we provided information about the aims and funding of the project, and assured them that responses would be treated confidentially in accordance with data protection regulations. We consciously chose a slightly different topic for the article than the topic of the questionnaire because we did not want to influence the respondents.

A link was provided that led to the SurveyMonkey platform. The criterion was that respondents should be between 18 and 50 years of age. The lower age limit was applied in order to avoid the need to obtain parental consent, and the upper age limit was applied because this is the end of natural fertility (Bunting et al., 2013, Eijkemans et al., 2014). The same age limit was used for men and women in order to avoid suggesting to the respondents that women’s fertile age is lower than men’s. Before respondents completed the questionnaire, they were asked to give consent. In total, 1582 respondents were recruited within 1 month. However, only those respondents who fully answered all of the questions were considered in this study, so the sample consisted of 1370 cases (dropout rate 13%). Data were exported from SurveyMonkey in a stata format. All answers for which the same IP address was used more than once (11 cases) were dropped. As no incentives were offered for completing the questionnaire, it was of no interest to the respondents to complete the questionnaire more than once. However, there were some drawbacks to not using incentives. People who were interested in this topic were more likely to fill out the questionnaire, so their level of knowledge was probably higher than average.

### Ethics

The study protocol was approved by the National Research, Development and Innovation Office (NKFI-PD-123789).

### Data analysis

Descriptive statistics were computed for each item (i.e. mean, standard deviation and proportion). Cronbach’s alpha was calculated to measure the reliability and internal consistency of the eight knowledge items. Pearson’s Chi-squared test was performed in order to investigate differences in frequencies between demographic categories [i.e. age group, sex, educational background, religiosity, number of children, intention to have (additional) children and previous experiences with ART] and by survey item. Analysis of variance was also performed to examine associations between each demographic variable and self-assessment knowledge level and actual knowledge level related to ART. Finally, linear regression was conducted to reveal the factors that can influence attitudes towards ART. The statistical analysis was conducted using Stata 13.0 (StataCorp, College Station, TX, USA).

## Results

### Characteristics of the study population

The mean ages of male and female participants were 38.9 and 37.1 years, respectively. The gender distribution was 58% female and 42% male. Higher-educated participants (at least a Master's degree) were over-represented (89.6%) in this sample. However, this does not contradict the objectives of this study, namely to examine knowledge about ART and attitudes towards ART among those who postpone their childbearing and potential users of ART. The rate of childless participants was 45.7%. Among the parents, 8% had one child, 52% had two children and 40% had more than two children. Among the respondents, 7% had already participated in ART treatment, and 23% had acquaintances who had previously undergone ART treatment. The majority (66.7%) of the study subjects were not religious or did not say whether they were religious or not, while 7.3% reported living their lives according to religious rules. The rate of those who were religious in their own way was 26%. All respondents who reported being religious were Christian.

### Self-rated knowledge and attitudes

Self-rated knowledge about general fertility was high, and this differed significantly by gender (*P* < 0.001). More than four-fifths of the respondents claimed that they were very or fairly knowledgeable about general fertility, and only approximately 1% of the respondents reported no knowledge about general fertility (see Table 1). Levels of self-rated knowledge about ART were lower among both genders compared with knowledge about general fertility. Again, women reported higher levels of knowledge than men (see Table 1). In spite of the difference in knowledge about general fertility and ART, there was a strong positive correlation between the two self-rating items (*r* = 0.52).

The distribution of attitudes towards ART did not differ significantly by gender. More than 80% of both men and women stated that they support ART or support it very strongly, while less than 3% of respondents stated that they oppose ART or oppose it very strongly (see Table 1).

### Specific dimensions of ART awareness

For the purpose of reporting the findings, ‘definitely not true’ and ‘probably not true’ responses were combined into one category, ‘not true’, while the ‘definitely true’ and ‘probably true’ categories were combined into ‘true’. Of the eight knowledge questions, 58.8% of the respondents answered only three questions correctly. As a scale, these knowledge items showed very low reliability or internal consistency (Cronbach’s alpha = 0.408). For four of the eight questions, the ‘do not know’ choice was the most common answer.

### Success rate and effectiveness of ART

Only 51.5% of the respondents provided the correct answer about the success rate of ART, while the rate of correct answers about the effectiveness of ART was much higher: for example, 74.9% of the participants knew that most women have to go through IVF more than once to have a baby (see Table 2). H1 was only partly confirmed. The second-highest rate of correct answers was observed in the case of egg-freezing, so H2 was rejected.

**Table 2:** Knowledge about assisted reproductive technology (ART) – item distribution, mean and standard deviation (SD).

Response Item	True or false	1 Much higher (%)	2 Somewhat higher (%)	3 Do not know (%)	4 Somewhat lower (%)	5 Much lower (%)	Mean	SD
1. Over 40 years of age, the success rate of fertility treatment among women is around 50%	F	0.4	5.3	42.8	25.1	26.4	2.28	0.93
2. The total cost of one cycle of in-vitro fertilization is under 500 Euro	F	31.4	31.4	34.8	2.1	0.3	3.92	0.87

	True or false	1 Definitely not true) (%)	2 Probably not true (%)	3 Do not know (%)	4 Probably true (%)	5 Definitely true (%)	Mean	SD

3. Most Hungarian fertility clinics will not provide treatment to women over 45 years of age	T	2.93	15.5	37.8	36.3	7.5	3.3	0.92
4. Egg-freezing before the age of 35 years can significantly prolong a woman’s fertility	T	1.4	6.4	20.1	55.2	16.9	3.8	0.84
5. The use of in-vitro fertilization poses health risks for a woman	T	8.9	29.1	15.7	27,6	18.7	3.18	1.28
6. Children conceived through the use of assisted reproductive technology have more long-term health problems than children conceived without the use of these fertility treatments	T	21.1	42.9	23.2	10.2	2.6	2.3	1
7. Most women have to go through in-vitro fertilization more than once to have a baby	T	2.2	7.2	15.7	52.4	22.5	3.86	0.92
8. The upper age limit for a man to be treated at most Hungarian fertility clinics is 55 years	F	1.9	7.6	69.2	19.8	1.4	3.11	0.63

*n* = 1370.

### Age limits for access to ART

Regarding the age limits for access to ART, 69.2% and 37.8% of the respondents did not know about the male and female age limits, respectively (see Table 2). Thus, H3 was confirmed (i.e. more people were aware of the age limit on women for access to ART at most Hungarian fertility clinics, but wrongly believed that there was an age limit for men as well).

### Risks of ART

The highest rate of incorrect answers was observed regarding the risks of ART for conceived children (64%) and for women (38%) (see Table 2). Consequently, H4 was confirmed (i.e. there is a lack of knowledge about the risks of ART).

### Cost of ART

Almost two-thirds of the participants gave correct answers about the cost of ART (see Table 2). This supports H5.

### Influence of sociodemographic variables on knowledge items

#### Gender

Women reported higher self-rated knowledge about ART (see Table 1). In parallel, the mean rate of correct answers for the actual knowledge items was 50.3% (8.8–76.6%) and 42% (10.1–73%) among women and men, respectively. Significant differences between genders were found in six of the eight items. The greatest difference between the two genders was found for the item concerning the risk of ART for women’s health: slightly more than half (51.5%) of male respondents did not know that IVF exposes women to health risks, compared with 28.1% of female respondents. Lack of knowledge was greatest regarding the risk of ART treatment for conceived children: only 14.1% of women and 10.5% of men were aware of this risk (see Table 2).

#### Education

Mean self-rated knowledge about ART was similar in the higher- and lower-educated subgroups (2.6 versus 2.5, respectively). The mean rate of correct answers was 50.3% (8.8–76.6%) and 42% (10.1–73%) in the two groups, respectively. Additionally, the actual knowledge of the higher-educated subgroup was significantly higher for two questions compared with the lower-educated group.

#### Age

Self-rated knowledge was higher in the older subgroup (age 36–50 years) than the younger subgroup (18–35 years): very knowledgeable (18.7% versus 8%, respectively) and fairly knowledgeable (38.4% versus 27.3%, respectively). Accordingly, the actual knowledge of the older subgroup was significantly higher for five questions compared with the younger group (see Table 3).

**Table 3:** Rate of correct answers by gender, education and age (%).

	Gender			Education			Age (years)		
Response item	Female	Male	*P*-value	Lower	Higher	*P*-value	18–35	36–50	*P*-value
1. Over 40 years of age, the success rate of fertility treatment among women is around 50%	54.8	47.1	0.008	49.7	53.7	0.02	45.9	54.9	0.000
2. The total cost of one cycle of in-vitro fertilization is under 500 Euro	67.6	56.7	0.000	60.4	65.6	NS	57.3	66.6	0.001
3. Most Hungarian fertility clinics will not provide treatment to women over 45 years of age	47.8	38.1	0.001	44.3	43.4	NS	39.8	46	0.007
4. Egg-freezing before the age of 35 years can significantly prolong a woman’s fertility	76.2	67.19	0.003	70.6	73.7	NS	71.4	73.1	NS
5. The use of in-vitro fertilization poses health risks for a woman	71.9	48.5	0.000	42.3	49.6	0.02	36.7	52.3	0.000
6. Children conceived through the use of assisted reproductive technology have more long-term health problems than children conceived without the use of these fertility treatments	14.1	10.5	0.03	11.9	13.3	NS	11.6	13.2	NS
7. Most women have to go through in-vitro fertilization more than once to have a baby	76.6	73	Ns	73.2	76.3	NS	70.2	77.9	0.000
8. The upper age limit for a man to be treated at most Hungarian fertility clinics is 55 years	8.8	10.1	Ns	8	14	NS	7.8	10.2	NS

NS, not significant.

Analysis of variance calculated on original data (five-point scale).

#### Other potential factors: Parenting status, previous ART experience and religiosity

There was a significant difference between parents and childless people: mean self-rated knowledge was higher among those who had children compared with their childless counterparts (2.7 versus 2.4, *P* < 0.001). Furthermore, there was a significantly higher level of knowledge among parents compared with their childless counterparts in the case of five actual knowledge questions (Items 2, 3, 5, 6 and 7). Respondents who had experience of ART treatment and those with acquaintances who had undergone ART treatment had higher mean self-rated knowledge than respondents who did not have any experience with ART treatment (3.8%, 2.8% and 2.4%, respectively, *P* < 0.001). Similarly, the highest actual knowledge was observed for all of the items among those who had experience of ART treatment, followed by those with acquaintances who had undergone ART treatment. The lowest rate was found among respondents who did not have any experience with ART treatment: the mean rates of correct answers were 61% (13–88%) and 51.1% (10.1–73%), respectively (*P* < 0.05). This supports H6.

#### Influence of sociodemographic variables on attitudes towards ART

A linear regression model was used to examine which factors can influence attitudes towards ART. All the sociodemographic variables were included (i.e. gender, education, age group, religiosity, number of children, and whether the participant had any previous experience with ART). Most of these sociodemographic variables did not influence attitudes towards ART, apart from religiosity (Table 4). Those respondents who classed themselves as religious were less likely to support ART compared with those who were not religious. This supports H7 regarding the negative effect of religiosity on attitudes. Besides religiosity, previous ART experience had a significant effect. Respondents who had any prior experience with ART treatments were more likely to support it. The strongest supporters of ART treatment were respondents who had a history of ART treatment, followed by those who had acquaintances who had undergone ART treatment compared with those who did not have any ART experience.

**Table 4:** Linear regression model of attitudes towards assisted reproductive technology (ART).

Dependent variable: Overall, to what extent do you support or oppose ART treatment?				
Independent variables	Categories	Coefficients	95% CI	
Gender	Men	Ref.	Ref.	Ref.
	Women	0.05	−0.03	0.13

Education	Lower education	Ref.	Ref.	Ref.
	Higher education	0.03	−0.03	0.09

Age category (years)	18–35	Ref.	Ref.	Ref.
	36–50	−0.01	−0.11	0.09

Religiosity	Not religious	Ref.	Ref.	Ref.
	Could not say whether religious or not	−0.06	−0.24	0.11
	Religious in their own way	−0.21***	−0.29	0.12
	Live their life according to religious rules	−0.91***	−1.07	0.09

ART experience	No	Ref.	Ref.	Ref.
	Acquaintances underwent ART	0.1*	0.01	0.2
	Personally underwent ART	0.4***	0.24	0.55

*n*	1370			
*r*^2^	0.12			

CI, confidence interval.

## Discussion

This research examined knowledge and attitudes about ART in Hungary. While there is significant existing research about fertility awareness (Bretherick et al., 2010, Bunting et al., 2013, Daniluk et al., 2012, Daniluk and Koert, 2013, Harper et al., 2017, Maheshwari et al., 2008, Meissner et al., 2016; Pedro et al., 2018), papers focusing on awareness of and attitudes towards ART are less widespread (Adashi et al., 2000, Bunting et al., 2013, Daniluk et al., 2012, Daniluk and Koert, 2013, Hashiloni-Dolev et al., 2011, Meissner et al., 2016, Stoebel-Richter et al., 2012). To the authors’ knowledge, this is the first study in Hungary to systematically examine the level of knowledge about ART among men and women. The findings show that self-rated knowledge about general fertility is higher than self-rated knowledge about ART, which might indicate that ART is not part of common knowledge and is less emphasized in the biology curriculum in primary and high schools in Hungary. These results are consistent with previous research conducted in Canada, Australia, the USA and European countries (Adashi et al., 2000, Cheung et al., 2019, Daniluk et al., 2012, Daniluk and Koert, 2013, Stoebel-Richter et al., 2012).

Regarding the actual knowledge items, approximately two-thirds of the male respondents (71.3% of childless men) and over half of the female respondents (60.5% of childless women) answered just three of eight items correctly. This indicated a higher level of knowledge than Daniluk et al., 2012, Daniluk and Koert, 2013 measured in a Canadian sample. These differences may be due to the different sample compositions: in the present sample, higher-educated people were over-represented. As well as childless men and women, the sample included parents, who seemed to have higher levels of knowledge. Furthermore, the present research was conducted in 2020, whereas the Canadian research was conducted 10 years previously; knowledge about ART may have become more pervasive over time due to social media and the increasing prevalence of ART (Shlomo and Kabizon-Pery, 2019, Präg and Mills, 2017).

There was a knowledge gap in more dimensions. Approximately half of the respondents (54.8% of women and 47.1% of men) were aware that the success rate of ART among women aged >40 years is <50%. According to the largest fertility clinics in Hungary, the success rate of women aged between 42 and 43 years was 8% for 2000–2008 (Kaáli Institute, 2019). Levels of knowledge about the effectiveness of ART were even lower. One-quarter of both men and women did not know that most women have to go through the IVF cycle more than once to have a baby. These results are consistent with previous research highlighting that although people are aware of the decline in fertility due to age, they still overestimate the chances of becoming pregnant, both spontaneously and through ART treatment (Adashi et al., 2000, Garcia et al., 2016, Hammarberg et al., 2013; Meissner et al., 2016, Peterson et al., 2012; Stoebel-Richter et al., 2012, Wyndham et al., 2012).

More than half of the respondents were aware of the costs of ART procedures; however, men were significantly less knowledgeable about this than women (43.3% versus 32.4% did not provide the correct answer, respectively). Knowledge of the cost of ART treatment is important because pervious research has found that its cost affects not only the usage of ART but also the number of embryos transferred (Chambers et al., 2013). Moreover, an even higher number of participants (76% of women and 67% of men) knew that social egg-freezing before 35 years of age can significantly prolong women’s fertility. This result was only slightly below the 83% found by Lallemant et al. (2016) among women in the UK and Denmark, where social egg-freezing is possible, unlike in Hungary (Keane et al., 2017, Sándor et al., 2018). It is most likely that people heard of social egg-freezing when global technology giants such as Apple and Facebook announced that they would pay for female employees to freeze their eggs for later reproductive use in 2014 (Mayes et al., 2018).

It was striking to note that the participants in this study largely underestimated the risks of ART. In total, 55% of the respondents (67% of men and 44% of women) did not know that IVF exposes women to health risks. The greatest gender difference was found for this item. Additionally, almost 90% of the Hungarian respondents did not know that ART can pose risks for the conceived children. These results are consistent with previous research: based on an American online survey (Fortin and Abele, 2016) conducted among women aged 24–49 years, only 17% of the respondents knew that the level of malformations is higher in children conceived via ART compared with children conceived spontaneously.

Furthermore, this analysis revealed that knowledge related to ART is not coherent, and it depends on sociodemographic factors such as age, gender, education level, having children and previous experience with ART procedures. As with most previous studies, this study found that women have higher levels of awareness about ART than men (Bunting et al., 2013, Meissner et al., 2016, Stoebel-Richter et al., 2012), which may be due to social norms supporting that childbearing is women’s responsibility. The present results are also consistent with previous studies in terms of higher education being associated with greater knowledge and fertility awareness (Bunting et al., 2013, Daniluk and Koert, 2013, Garcia et al., 2016, Meissner et al., 2016), probably because more-educated respondents seek more information (Pedro et al., 2018). Respondents in the older group were closer to their end of the reproductive life span, and thus may have been more motivated to seek information and be more aware about general fertility and ART-related fertility issues (Bunting et al., 2013; Pedro et al., 2018). Parents and those with some ART experience were more knowledgeable than childless people and those without any ART experience. These results confirm the findings of previous research (Maeda et al., 2015).

Regarding attitudes, the study respondents had positive attitudes towards ART treatment: 90.4% of the participants support it or support it very strongly. No significant differences were found between sociodemographic groups (gender, educational level and age). The small *r*^2^ value in the regression models also indicates that other factors, such as sociodemographic variables, cannot influence attitudes towards ART. These results are consistent with previous research that highlights the importance of norms and values instead of sociodemographic variables in attitudes towards ART (Bunting et al., 2013, Präg and Mills, 2017, Szalma and Djundeva, 2019). However, this model found that two factors significantly influence attitudes to ART: religiosity and previous experience with ART. Respondents who classed themselves as more religious were less supportive of ART. This result confirms previous research (Chilaoutakis et al., 2002, Shreffler et al., 2010, Szalma and Djundeva, 2019). At the same time, respondents who had undergone ART treatment previously were the most supportive. Previous studies revealed that respondents who had experienced infertility were more supportive of ART (Shreffler et al., 2010) because they were likely to have a greater degree of understanding and interest in treatments. Furthermore, if the respondents had acquaintances who had undergone ART treatment, they were more likely to support ART compared with respondents who did not have any previous ART experience.

## Conclusion

As the number of ART users is expected to increase significantly in Hungary as the Government has made it free of charge, it is important for potential users to be properly informed about the related risks among those of reproductive age, and its effectiveness and success rate for those of advanced age in order to avoid involuntary childlessness. A clear message would be that ART can help most couples facing infertility problems to have a baby if the woman is aged <30 years, but it is unlikely they will have a baby if the woman is aged >40 years (Utting and Bewley, 2011). Doctors have a unique opportunity to communicate with women about their reproductive life span, but public health programmes would be more effective than individual consultations. That is why a broad-based approach is needed to make factual and accessible information available for those of reproductive age in Hungary. Another potential method to increase knowledge among the Hungarian population is to include ART in the biology curriculum in secondary schools.

### Limitations and further research

The findings are limited because the study participants were recruited via an online survey. Therefore, higher-educated men and women were over-represented in the sample. However, this population is more likely to delay childbearing, meaning that these people probably have a greater need for ART treatment. Moreover, those who completed the questionnaire may have had a greater interest in fertility and ART-related issues than those who did not complete the questionnaire. However, it should be noted that the online survey was conducted over 1 month (February 2019), which represents a strength of the online survey, as external factors had less effect on the results. Another strength of an online survey design compared with a face-to-face survey design was that sensitive knowledge questions could be asked in such a way that the respondents did not experience this as failure in front of the interviewer if they did not know the correct answers. They could choose ‘do not know’ without fear of contempt if they were uncertain. However, further research is needed in order to examine people’s ART-related knowledge in a representative sample in Hungary, and also to measure changes in knowledge and behaviour of women and men of reproductive age further to the newly introduced ART regulations in Hungary. It would also be interesting to measure if the number of ART users increases or not now it is freely available for Hungarians. Based on this research, it is recommended that further research focusing on either ART-related knowledge or attitudes towards ART should include not only sociodemographic questions but also ART-related experience (personal or via an acquaintance) as this can have a large effect. However, this variable may have had a very strong effect in this study because respondents with some ART experience were over-represented in the study sample.

## Declaration

The authors report no financial or commercial conflicts of interest.
