# Supplementary material for: Knowledge and attitudes about assisted reproductive technology: Findings from a Hungarian online survey
Source: Reprod Biomed Soc Online. 2021 Jul 3;13:75–84. doi: 10.1016/j.rbms.2021.06.005 (PMC8340049; doi:10.1016/j.rbms.2021.06.005)
Supplement: Supplementary data 1 [file mmc1.docx]

Dear Participants,

This research project, entitled ‘Fertility Knowledge and Knowledge Transfer Effectiveness’ (NKFIH 123789) is supported by the National Office for Research, Development and Innovation. The aim of this research is to measure the knowledge and attitudes related to fertility and assisted reproduction among men and women aged between 18 and 50.

The questionnaire is completely anonymous and takes only 5–10 minutes to complete. Your name is not required, and no one can identify individual answers in this study.

You also have the right to terminate the survey at any time prior to submission.

If you complete and submit this survey, it will be assumed that you have consented to participating in this study. When you have completed the survey, please be sure to click on the SUBMIT button, or your responses will be lost.

We welcome everyone’s participation who is between 18 and 50 and thank you for contributing to the success of our research! Your responses will be treated confidentially in accordance with data protection regulations.

If you have any questions related to this research, you can send it to the following e-mail address:

Szalma Ivett

szalma.ivett@tk.mta.hu

Part 1

1. Overall, how would you rate your current knowledge of fertility**?**

No knowledge

Some knowledge

Fairly knowledgeable

Very knowledgeable

2. Overall, how would you rate your current knowledge of assisted human reproduction procedures and fertility treatments?

No knowledge

Some knowledge

Fairly knowledgeable

Very knowledgeable

3. What do you think about assisted human reproduction procedures and fertility treatments?

Completely opposed

Rather oppose

Neither oppose either support it

Rather support

Completely support

Part 2

1. For women over 30, overall health and fitness level is a better indicator of fertility than age.

Definitely not

Probably not

Uncertain

Probably

Definitely

2. Taking birth control pills for more than 5 years negatively affects a woman's fertility.

Definitely not

Probably not

Uncertain

Probably

Definitely

3. A woman’s eggs are as old as she is.

Definitely not

Probably not

Uncertain

Probably

Definitely

4. Over 40 years, the success rate of fertility treatment among women is around 50%.

Much lower

Somewhat lower

Do not know

Somewhat higher

Much higher

5. The total cost of one cycle of in-vitro fertilization is under 150 thousand HUF.

Much lower

Somewhat lower

Do not know

Somewhat higher

Much higher

6. There is a progressive decrease in a woman's ability to become pregnant after the age of 35.

Definitely not

Probably not

Do not know

Probably

Definitely

7. The rates of miscarriage are significantly higher for women in their 40s than for women in their 30s, even for physically fit women in excellent health.

Definitely not

Probably not

Do not know

Probably

Definitely

8. Most Hungarian fertility clinics will not provide treatment to women over the age of 45.

Definitely not

Probably not

Do not know

Probably

Definitely

9. Egg-freezing before the age of 35 can significantly prolong a woman’s fertility.

Definitely not

Probably not

Do not know

Probably

Definitely

10. Sexually transmitted diseases (e.g. chlamydia, gonorrhoea) significantly increase the risk of later infertility.

Definitely not

Probably not

Do not know

Probably

Definitely

11. The age of her male partner is an important factor in a woman’s chances of becoming pregnant.

Definitely not

Probably not

Do not know

Probably

Definitely

12. The use of in-vitro fertilization poses health risks for a woman.

Definitely not

Probably not

Do not know

Probably

Definitely

13. Children conceived using assisted reproductive technology have more long-term health problems than children conceived without the use of these fertility treatments.

Definitely not

Probably not

Do not know

Probably

Definitely

14. Most fertility conditions are caused by problems with the woman’s fertility.

Definitely not

Probably not

Do not know

Probably

Definitely

15. Most women have to go through IVF more than once to have a baby.

Definitely not

Probably not

Do not know

Probably

Definitely

16. A woman's too low bodyweight affects her chances of conceiving a child.

Definitely not

Probably not

Do not know

Probably

Definitely

17. The upper age limit for a man to be treated at most Hungarian fertility clinics is 55.

Definitely not

Probably not

Do not know

Probably

Definitely

18. The quality of a man’s sperm decreases significantly after the age of 50.

Definitely not

Probably not

Do not know

Probably

Definitely

19. Smoking cigarettes or marijuana can reduce the quality of a man’s sperm.

Definitely not

Probably not

Do not know

Probably

Definitely

20. Children born to fathers older than 45 have higher rates of learning disabilities, autism, schizophrenia and some forms of cancer.

Definitely not

Probably not

Do not know

Probably

Definitely

21. Excessive alcohol consumption can increase infertility among both women and men.

Definitely not

Probably not

Do not know

Probably

Definitely

22. Smoking cigarettes or marijuana negatively affects a woman's fertility.

Definitely not

Probably not

Do not know

Probably

Definitely

23. Frequent use of hot water baths and/or saunas can damage sperms.

Definitely not

Probably not

Do not know

Probably

Definitely

24. If a man takes steroids, his ability to fertilize decreases.

Definitely not

Probably not

Do not know

Probably

Definitely

25. A woman's too high bodyweight affects her chances of conceiving a child.

Definitely not

Probably not

Do not know

Probably

Definitely

Part 3

1. What is your gender?

2 What year were you born?

|  |  |  |
| --- | --- | --- |

3. Do you have any biological children?

4. Which of the following statements best describes you?

1) Religious, live my life according to religious rules.

2) Religious in my own way.

3) Could not say

4) Not religious.

5. What is the highest grade or level of education that you have completed?

6. Have you already participated in ART treatment?

Yes

No

I have acquaintances who underwent such treatment

Do you have any comments on this questionnaire or about this research?
